# Integrated analysis of MIOX gene in prognosis of clear-cell renal cell carcinoma

**DOI:** 10.1038/s41419-025-07698-7

**Published:** 2025-05-08

**Authors:** Yiqiu Wang, Jiayi Lu, Bohan Lin, Jiayin Chen, Fei Lin, Qingshui Zheng, Xueyi Xue, Yong Wei, Shaohao Chen, Ning Xu

**Affiliations:** 1 Department of Urology, Urology Research Institute, The First Affiliated Hospital, Fujian Medical University, Fuzhou, China; 2 Department of Urology, National Regional Medical Center, Binhai Campus of the First Affiliated Hospital, Fujian Medical University, Fuzhou, China; 3 Department of Urology, Renji Hospital, Shanghai Jiao Tong University School of Medicine, Shanghai, China

**Keywords:** Urological cancer, Cancer genomics

## Abstract

Clear-cell renal cell carcinoma (ccRCC) is a highly aggressive malignancy that originates in the kidney. It often exhibits a limited response or can be refractory to a wide range of anti-cancer therapies, including tyrosine kinase inhibitors (TKIs) and immune checkpoint inhibitors. Ferroptosis is a form of oxidative, iron-dependent cell death characterized by lipid peroxidation. Targeting ferroptosis may offer a promising alternative therapeutic strategy for cancer cells that are resistant to existing treatments. The impact of ferroptosis-related genes on the prognosis of ccRCC patients is still not fully understood. In this study, we identified 30 differentially expressed ferroptosis-related genes in ccRCC samples compared to normal tissues using data from The Cancer Genome Atlas (TCGA). Lasso regression analyses, along with Kaplan-Meier analysis, were conducted to identify genes associated with prognosis. Based on scRNA-seq and spatial transcriptome analysis, we identified specificity of MIOX in ccRCC. Furthermore, MIOX demonstrated the highest significance, highlighting its independent prognostic value as a single gene in ccRCC. Our findings suggest that MIOX could serve as potential targets for therapeutic interventions in ccRCC.

## Introduction

Renal cell carcinoma (RCC), a common cancer arising from the renal epithelium, was reported to have 431,288 new cases and 179,368 deaths worldwide in 2020. Among these, about 271,249 cases were identified in men, while 139,756 were found in women [1]. Clear-cell renal cell carcinoma (ccRCC) is the predominant histological variant of RCC, representing approximately 75% of all cases [2]. Individuals diagnosed with ccRCC generally face a poorer prognosis compared to those with other histological forms of RCC [3].

Ferroptosis is an emerging form of regulated cell death characterized by iron-dependent lipid peroxidation [4]. Unlike apoptosis, ferroptosis involves the accumulation of lipid reactive oxygen species (L-ROS) when their levels surpass the antioxidant capacity of glutathione-dependent peroxidase (GPX4), resulting in a disruption of cellular redox balance [5]. Ferroptosis is characterized by three key features: (i) the oxidation of polyunsaturated fatty acids (PUFAs) in membrane phospholipids; (ii) the presence of redox-active iron; and (iii) a diminished ability to repair lipid hydroperoxides (LOOH) [6]. In cancer cells, elevated levels of iron and reactive oxygen species (ROS) are typically observed, which support their metabolic activity and proliferation [7]. From this viewpoint, targeting ferroptosis could offer a promising alternative treatment strategy for cancer cells that are resistant to conventional chemotherapy or molecular-targeted therapies.

Cystine uptake can be inhibited by the small molecule inhibitor BSO, or the synthesis of γ-glutamylcysteine can be inhibited by the small molecule inhibitor erastin, thereby suppressing GSH biosynthesis in ccRCC cell lines and triggering ferroptosis [8]. Another lipid analysis revealed a significant increase in triglycerides and cholesteryl esters in ccRCC tumor tissues, suggesting that renal cancer cells may be particularly sensitive to ferroptosis [9]. Ferroptosis may offer new therapeutic targets. The relationship between ferroptosis and ccRCC has not been thoroughly studied, and some genes related to ferroptosis remain unexplored in their role within ccRCC.

In this study, we analyzed ferroptosis-related differential genes with prognostic value from the TCGA-KIRC cohort and explored their biological functions in ferroptosis in clear-cell carcinoma, correlating them with previously reported genes associated with ferroptosis in ccRCC.

We found that silencing MIOX at the in vitro level, rather than other candidate genes, significantly affects the malignant phenotype of ccRCC. Myo-inositol oxygenase (MIOX) is a 33 kDa non-heme ferritin that converts inositol into D-glucuronic acid through the glucuronic acid-xylose pathway [10]. Its transcription is influenced by factors such as oxidative stress, free fatty acids, and elevated glucose levels [11]. Increased levels of MIOX lead to enhanced production of reactive oxygen species (ROS) while diminishing the availability of reduced nicotinamide adenine dinucleotide phosphate (NADPH) and glutathione (GSH), ultimately lowering the cell’s antioxidant defense [12]. MIOX is particularly abundant in the renal tissues, where it plays a crucial role in metabolic processes. lower levels of MIOX can be found in other tissues, but its primary roles and regulatory functions are most pronounced in the kidney and liver.Further exploration of the role of MIOX in the treatment of kidney cancer has significant clinical implications.

## Results

### Identification of 30 ferroptosis-related DEGs

**Fig. 1:**
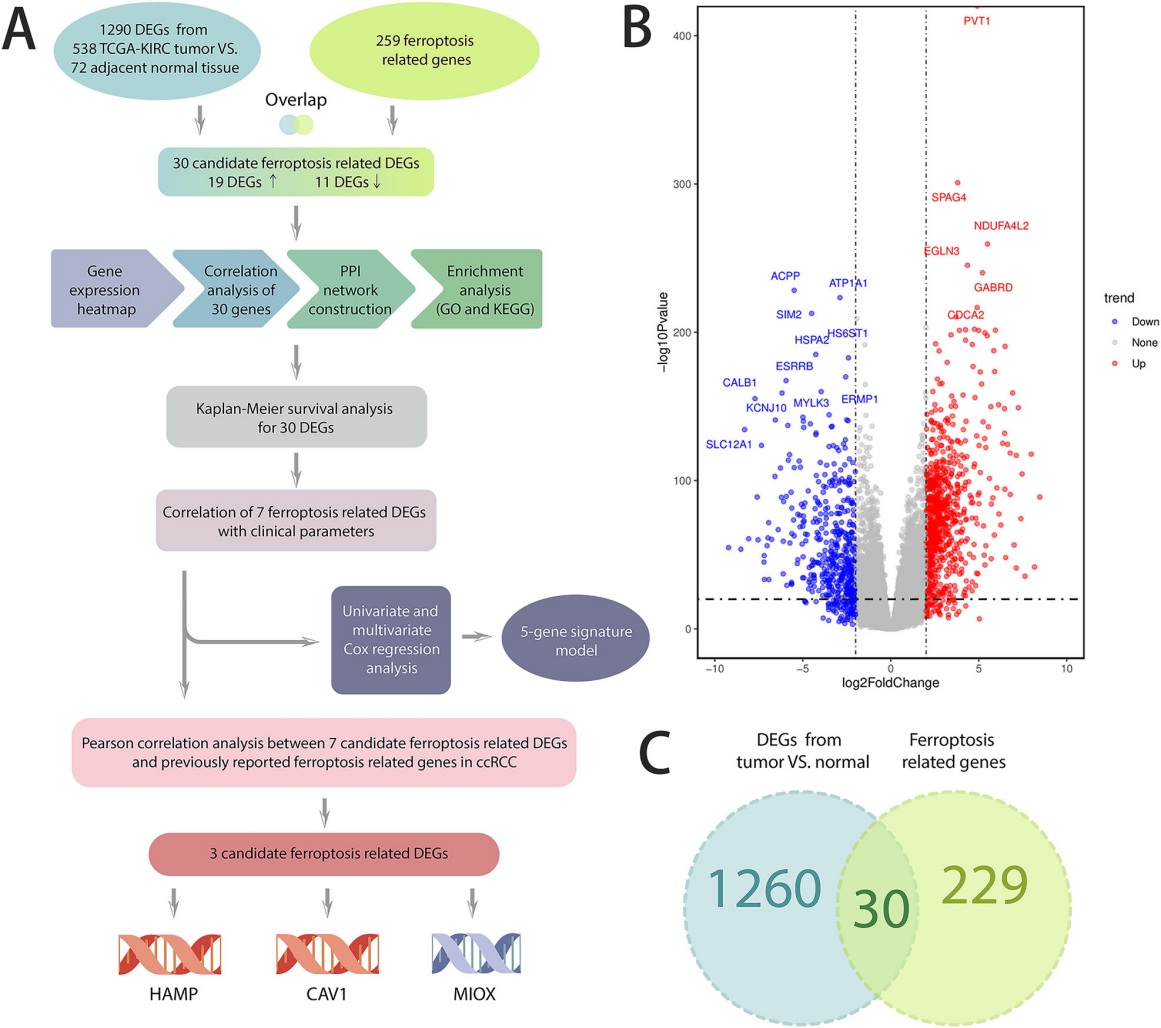
Identification of 30 ferroptosis-related DEGs. **A** Flowchart of the study; **B** Volcano plot for DEGs between TCGA-KIRC tumors and adjacent normal tissues; **C** Venn diagram to identify ferroptosis related DEGs.

To provide a systematic overview of our study, we created a flowchart (Fig. 1A). The differential gene analysis conducted between 538 TCGA-KIRC tumor samples and 72 adjacent non-tumor samples identified a total of 1290 differentially expressed genes (DEGs). Among these, 802 genes were found to be upregulated, while 408 were downregulated (Fig. 1B). As illustrated in the Venn diagram (Fig. 1C), we identified 30 candidate ferroptosis-related DEGs through the intersection of the 1290 DEGs and 291 known ferroptosis-related genes. Detailed information regarding these the 291 ferroptosis-related gene sets is presented in Table 1. Notably, all 30 candidate genes exhibited differential expression between clear-cell renal cell carcinoma tissues and adjacent non-tumorous tissues, with the exception of the ALB gene (Supplementary Fig. 1).

**Table 1:** Information of RCC organoid.

Sample type	Age	Sex	Sample source	Sample collection	Histological type	Sample ID
Primary tumor	53	M	Kidney (left)	Partial nephrectomy	ccRCC	KC-1
Primary tumor	76	F	Kidney (left)	Radical nephrectomy	ccRCC	KC-2
Primary tumor	65	M	Kidney (right)	Radical nephrectomy	ccRCC	KC-3

### Functional enrichment analysis of DEGs and PPI network construction

**Fig. 2:**
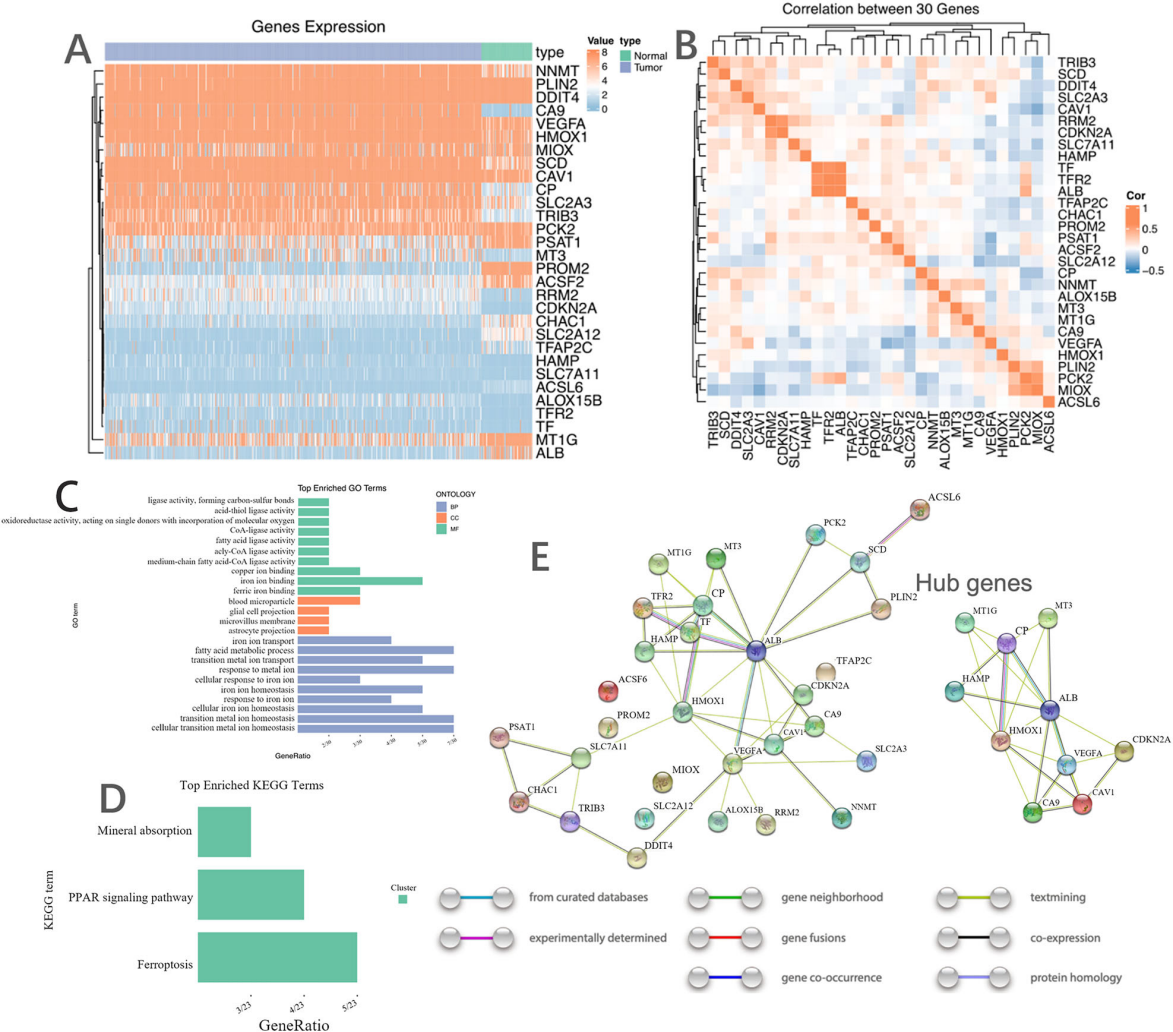
Functional enrichment analysis of 30-DEGs and PPI network construction. **A** Heatmap showing gene expression level in tumor and normal tissues; **B** Correlation analysis for 30 genes; **C** GO analysis items; **D** KEGG pathway; **E** PPI network constructed by STRING and Hub genes.

The heatmap in Fig. 2A illustrates the expression levels of the 30 candidate ferroptosis-related DEGs. Correlation analysis (Fig. 2B) among these genes reveals a strong association between TF, TFR2, and ALB.

To further explore the biological characteristics of these 30 candidate genes, we conducted Gene Ontology (GO) term and KEGG pathway analyses. The primary biological processes (BPs) identified include cellular transition metal ion homeostasis, response to metal ions, fatty acid metabolic processes, response to hypoxia, regulation of endocytosis, regulation of transcription from RNA polymerase II promoters in response to stress, and intrinsic apoptotic signaling pathways (Fig. 2C). The cellular component (CC) terminology highlights astrocyte projections, microvillus membranes, glial cell projections, and blood microparticles (Fig. 2C). The most prevalent molecular functions (MF) include ferric iron binding, iron ion binding, medium-chain fatty acid-CoA ligase activity, and acyl-CoA ligase activity (Fig. 2C).

The top enriched KEGG pathways encompass mineral absorption, the PPAR signaling pathway, and ferroptosis (Fig. 2D). We also constructed a protein-protein interaction (PPI) network using STRING for the 30 candidate genes (Fig. 2E). This interaction network suggests that MT1G, MT3, CP, HAMP, ALB, HMOX1, VEGFA, CDKN2A, CA9, and CAV1 may serve as hub genes (Fig. 2E).

### Prognostic significance and predictive efficacy of ferroptosis-related genes

**Fig. 3:**
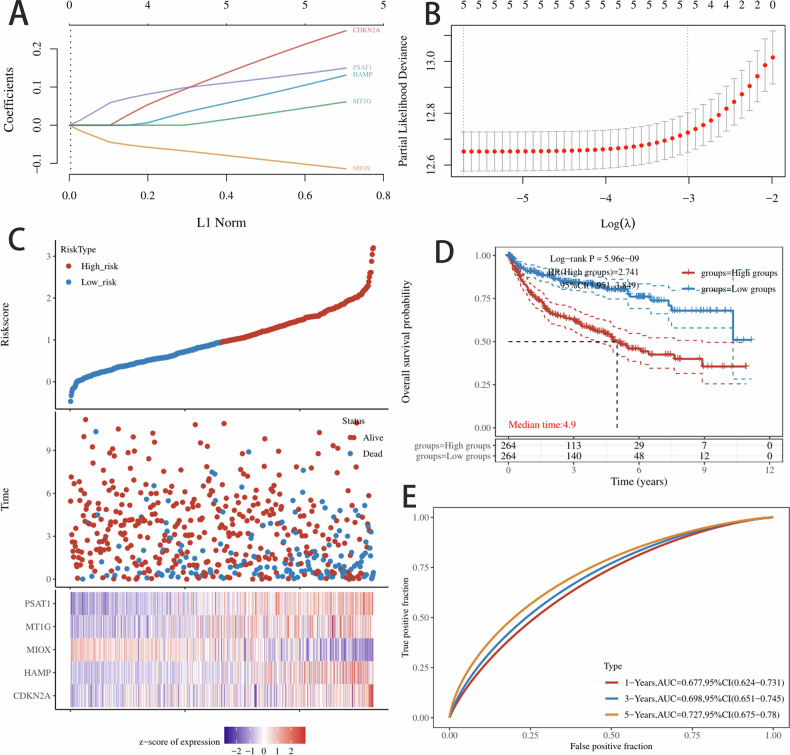
Establishment of the least absolute shrinkage and selection operator (LASSO) regression risk model. **A**, **B** The coefficients of selected features are shown by lambda parameter. The abscissa represents the value of lambda, and the ordinate represents the coefficients of the independent variable; **C** The Riskscore, survival time and survival status of selected dataset. The top scatterplot represents the Riskscore from low to high. Different colors represent different groups. The scatter plot distribution represents the Riskscore of different samples correspond to the survival time and survival status. The bottom heatmap is the gene expression from the signature; **D**, **E** Kaplan–Meier survival analysis of the risk model from dataset, comparison among different groups was made by log-rank test. HR (High exp) represents the hazard ratio of the low-expression sample relatives to the high-expression sample. The higher values of AUC corresponding to higher predictive power. HR > 1 indicates the gene is a risk factor, and HR < 1 indicates the gene is a protective factor. HR (95% Cl), the median survival time (LT50) for different groups, in years.

To investigate the relationship between gene expression and prognosis, we performed survival analysis on the 30 candidate genes based on their expression levels within the cohort. Proportional hazards model was used to identify 5 survival-related genes (PSAT1, MT1G, MIOX, HAMP and CDKN2A) in TCGA dataset (Supplementary Fig. 2). Lasso regression was employed to screen parameters, with the variation in the coefficients of these variables illustrated (Fig. 3A). Utilizing tenfold cross-validation for iterative analysis, we identified a model that demonstrated excellent performance with the fewest variables when λ was set (Log λ = −1.20). (Fig. 3B). We calculated the risk score of the TCGA dataset and determined the risk score distribution, showing that the higher the risk score and mortality rate of patients with the lower gene expression of selected five genes (Fig. 3C, D). Calibration curves show the predictive efficacy of the nomogram model at 1-, 3-, and 5-year intervals (Fig. 3E).

### MIOX signature in RCC via single-cell sequencing and spatial transcriptome

**Fig. 4:**
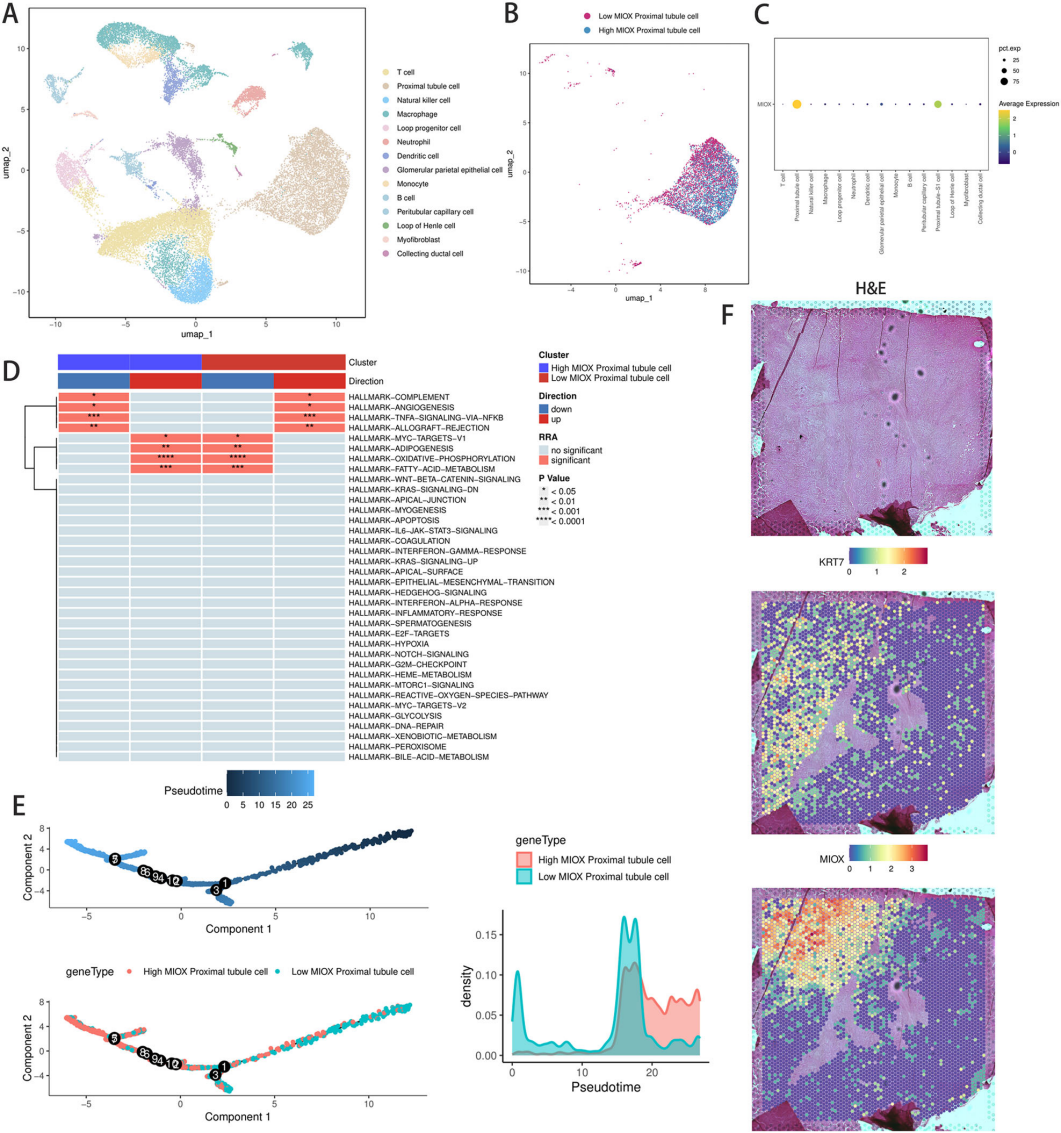
Single-cell RNA sequencing and spatial transcriptomics of ccRCC. **A** Single cell of three tumor samples and three adjacent normal samples of ccRCC in scRNA-seq database was classified according to cell types; **B** UMAP showing reclustering of tumor cells with different MIOX expression; **C** MIOX expression level was identified in different cell types; **D** Heat map representation of the GSVA showing specific hallmarks enriched in different group; **E** Distribution of cells in each cluster on the pseudo-time trajectory. Pseudo-time trajectory of single-cell transcriptomics data colored according to cluster. Cells were distributed along the main stem; **F** Spatial plots showing prediction scores of MIOX in tumor region, identified by H&E staining (GEO database, sample ID: GSM5924030, human RCC tissue) and KRT7 expression (bottom).

Additionally, single-cell transcriptomics was utilized to thoroughly investigate the gene expression profiles of specific cellular components within RCC tissues and their interactions. An analysis of GSE222703 revealed 14 cell clusters and 7 medium cell types (Fig. 4A). MIOX was highly expressed in proximal tubule cells (Fig. 4B, C). A heatmap representation of the GSVA highlights specific hallmarks enriched in the High-MIOX and Low-MIOX groups. It can be seen that the Low-MIOX expression group is significantly enriched in pathways related to complement, angiogenesis, and TNF-NFkB (Fig. 4D). Determination of cell trajectories and pseudotime distributions of RCC cells was carried out based on GSM5924030 and revealing existence of two cellular states during malignant cell development (Fig. 4E). As depicted, lower MIOX expression was not prominently existed within the core region of tumor (identified by KRT7) but in tumor-adjacent tissue (Fig. 4F).

### MIOX was required for RCC progression

**Fig. 5:**
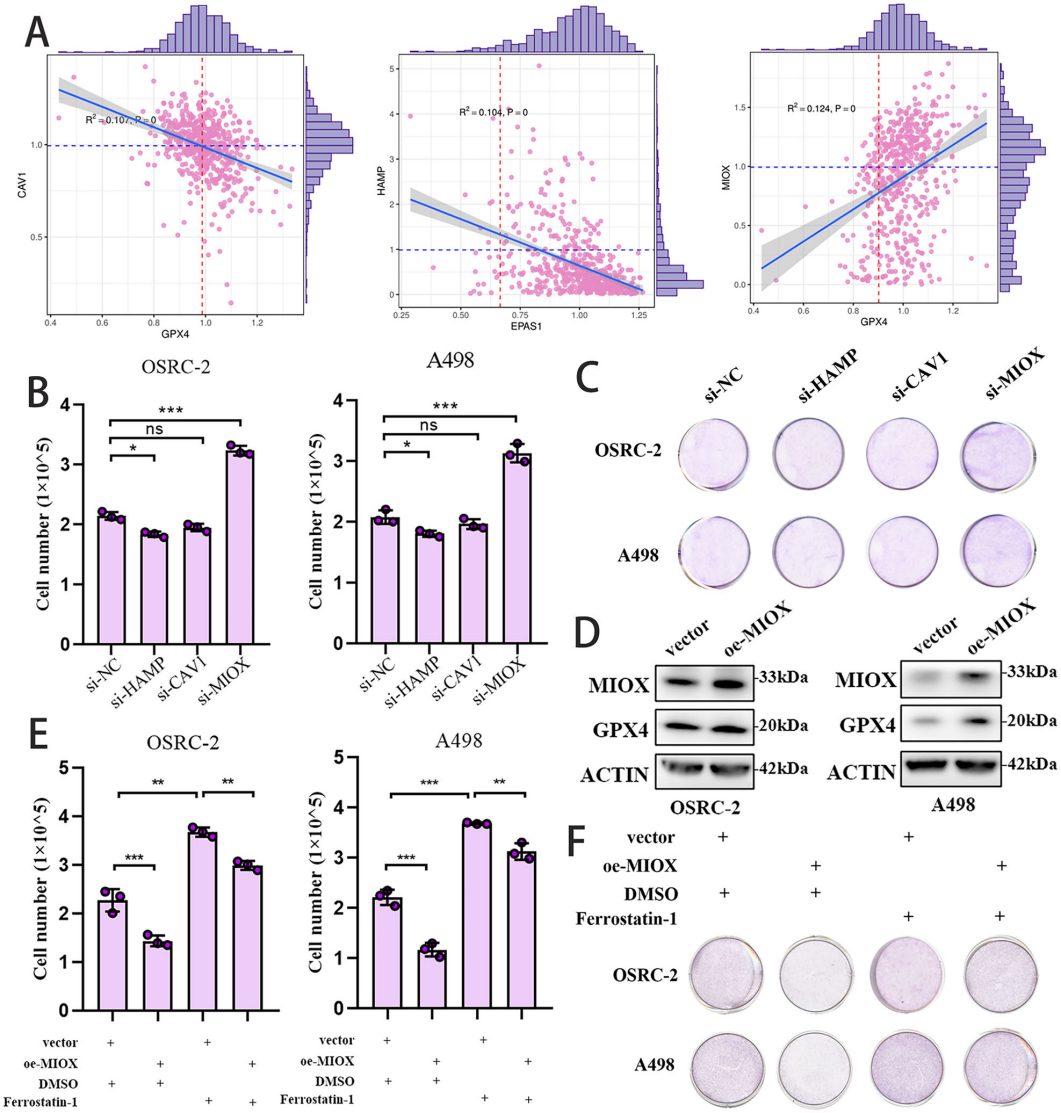
MIOX Inhibited ccRCC progression. **A** Correlation analyses conducted between three candidate genes and the expression of ferroptosis-related genes; **B**, **C** Silence of MIOX enhanced the capacity of tumor progression. Cell counting method to assess the proliferative efficiency of cells in a 384-well plate (**B**), Crystal violet staining method to assess the proliferative efficiency of renal cancer cells in a 12-well plate (**C**); **D** Efficiency of MIOX Overexpression was confirmed by western blot; **E**, **F** Tumor progression was measured in RCC cells bearing vector or oe-MIOX without and with the addition of Ferrostatin-1. Cell counting method to assess the proliferative efficiency of cells in a 384-well plate (**E**), Crystal violet staining method to assess the proliferative efficiency of renal cancer cells in a 12-well plate (**F**).

We conducted a correlation analysis between the seven candidate genes and publicly reported ferroptosis-related genes in clear-cell renal cell carcinoma (ccRCC). The analysis revealed a negative correlation between CAV1 and GPX4, as well as between HAMP and EPAS1. In contrast, MIOX exhibited a positive correlation with GPX4 (Fig. 5A). To explore the function of these three candidate genes in RCC cells, we transfected siRNA into two RCC cell lines. Unlike other candidates, inhibiting MIOX resulted in increased RCC proliferation (Fig. 5B, C). The efficiency of MIOX silencing was validated by western blot (Fig. S4A). Using transcriptomic data from different cancer types in the TCGA database for survival analysis, we observed that the expression differences of MIOX were not significantly associated with prognosis in other cancers, which suggests a certain degree of specificity of MIOX in RCC (Supplementary Fig. 3). We also created stable RCC cell lines with MIOX overexpression (Fig. 5D). Subsequent assays revealed that treatment with Ferrostatin-1 could partially counteract the proliferation induced by MIOX overexpression (Fig. 5E, F).

### MIOX was identified as a treatment target for ccRCC

**Fig. 6:**
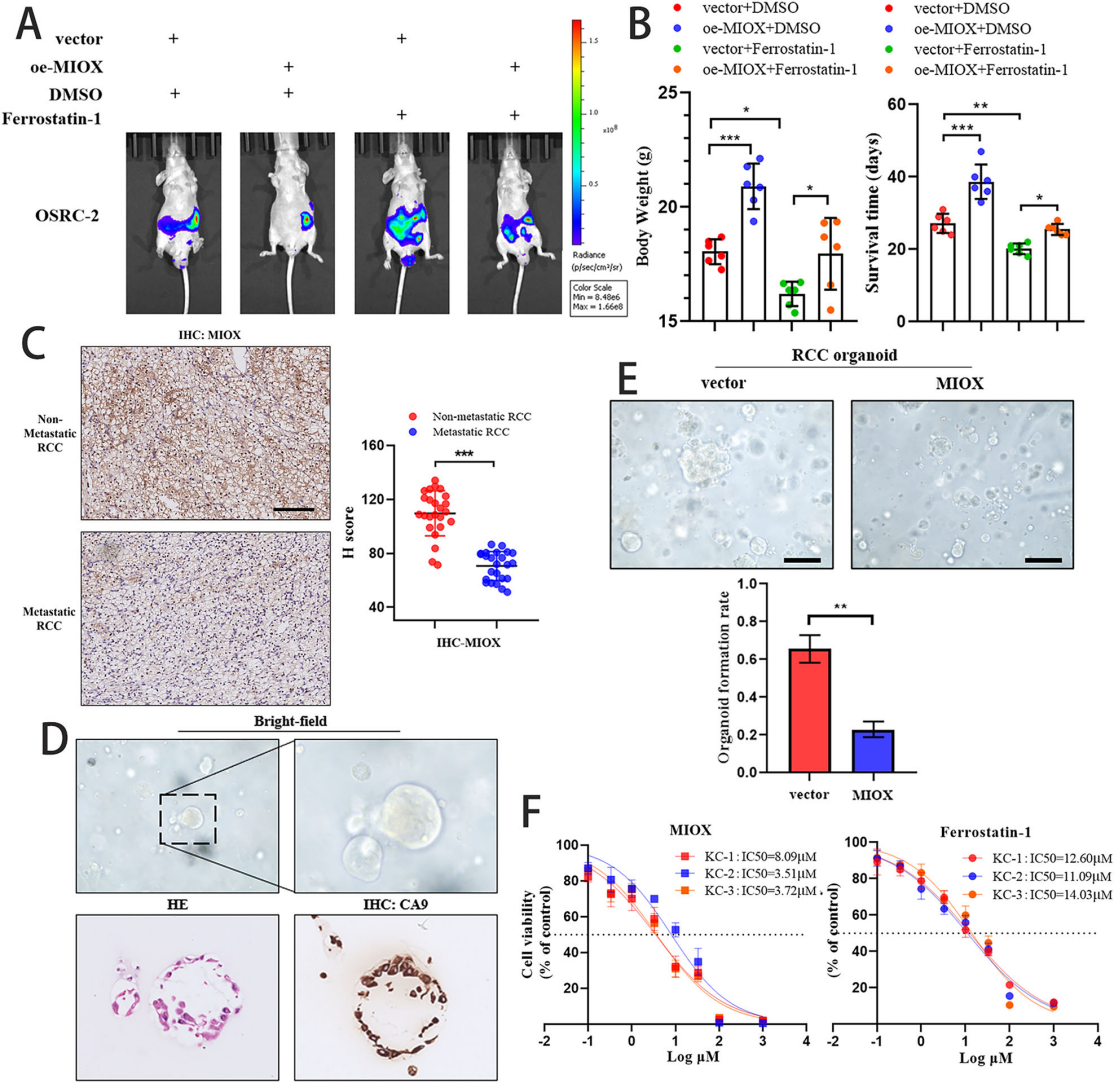
MIOX was identified as a treatment target for ccRCC. **A**, **B** Nude mice were orthotopically xenografted with luciferase-expressing cells, bioluminescent imaging was used to observe tumor progression; **C** IHC staining for MIOX in RCC patients, quantitative analysis of IHC staining was shown; **D** Representative image of H&E (Organoid section from RCC patients in First-Affiliated Hospital of Fujian Medical University) and IHC staining derived from established RCC organoids; **E** RCC organoids with MIOX overexpression expanded to almost twice the size of the control organoids; scale bars = 20 μm; **F** Dose–response curves of MIOX overexpression and ferrostatin-1 for organoid cultures from patients.

Following the subcutaneous implantation of luciferase-expressing RCC cells into the left kidneys of nude mice, we noted reduced tumor progression in the MIOX overexpression group compared to the control group (Fig. 6A, B). Immunohistochemical analysis of RCC tissue microarrays revealed a significant decrease in MIOX levels in metastatic RCC compared to non-metastatic RCC (Fig. 6C). We utilized qRT-PCR to assess the expression levels of MIOX in clinical samples and attempted to compare MIOX levels between the Non-Metastatic RCC group and Metastatic RCC group. Statistical analysis revealed MIOX expression was higher in Non-Metastatic RCC than Metastatic RCC (Fig. S4B). This result is consistent with IHC. We established three organoids sourced from different regions of tumors in RCC patients (Fig. 6D). To validate our findings, we examined the impact of MIOX overexpression in organoid cultures from RCC patients (K1, K2, and K3), which confirmed that MIOX exerts a suppressive effect on RCC (Fig. 6E, F). These findings collectively indicated that MIOX was upregulated in RCC tissues and acted as a suppressor of tumor progression in RCC.

## Discussion

The high recurrence rate of renal clear-cell carcinoma poses a significant challenge to current clinical treatment. For patients with metastatic or advanced renal clear-cell carcinoma, immune-targeted combination therapy is administered as the first-line treatment [13, 14]; however, the low drug response rate and resistance issues still need to be addressed. The current treatment dilemma lies in the fact that immune and targeted therapies can only provide patients with short-term effects and limited survival benefits. Therefore, identifying new therapeutic mechanisms is crucial for the treatment of renal clear-cell carcinoma.

Ferroptosis was proposed in 2012, and current research indicates that many cancer cells that are resistant to conventional chemotherapy or immunotherapy are more sensitive to ferroptosis [15, 16]. The high susceptibility of renal clear-cell carcinoma to ferroptosis is evident; however, there is limited research on the relationship between ferroptosis-related genes and renal clear-cell carcinoma. Many unknowns still exist regarding the determinants of this susceptibility. This study aims to determine whether ferroptosis-related genes play a prognostic role in renal clear-cell carcinoma, providing new directions for its treatment.

MIOX (myo-inositol oxygenase) is an enzyme found in the proximal tubules [17]. In cisplatin-induced acute kidney injury (AKI), MIOX has been reported to accelerate iron elevation, worsening tubular damage [18]. However, whether overexpression of MIOX can exacerbate renal cancer cell death remains a mystery. This study is the first to comprehensively analyze the role of MIOX genes in the progression, prognosis, and potential therapeutic value in ccRCC. We identified a novel prognostic panel of five ferroptosis signature genes and developed a robust risk model for predicting prognosis in ccRCC. Validation experiments through knockdown and overexpression in vitro and in vivo revealed that MIOX functions as a suppressor of tumor progression in RCC.

Regarding other candidate genes, such as HAMP and CAV1, although numerous studies have demonstrated their potential roles in RCC and their adverse effects on the prognosis of ccRCC patients [19, 20], their impact on the malignant phenotype of renal cancer cells in our in vitro model was not significant.

Low-expression markers provide important breakthroughs for tumor treatment. Inactivation of specific pathways (such as the p53 pathway) is important for tumor progression. Lower-expressed tumor suppressor genes, such as PTEN, are associated with activation of the PI3K/AKT/mTOR pathway, which has become key therapy for various tumors. Investigating the role of low-expression genes in key pathways aids in discovering new drug targets.

MIOX increased ROS production and reduced intracellular NADPH and GSH levels, leading to enhanced erastin- and RSL3-induced ferroptosis [21].

Since lower expression of MIOX is correlated with higher mortality, in this study, we demonstrated that high MIOX expression suppress tumor progression though inducing ferroptosis. Therefore, other ferroptosis inducers shall be tried to treat patients selected by the risk score proposed in this study.

However, overexpression of MIOX exacerbates the pathogenesis of cisplatin-induced AKI. It is crucial to avoid inducing AKI as a side effect. Although low-expression genes cannot be directly targeted to restore their expression, indirect interventions can be employed, such as activating downstream compensatory pathways to restore critical functions, reversing the silencing of low-expression genes through epigenetic modifications (e.g., demethylating agents), or directly restoring the function of target genes via gene therapy. Therefore, it is necessary to explore the specific mechanism of low MIOX gene expression.

In recent years, advancements in single-cell sequencing and spatial transcriptomics have led to significant breakthroughs in tumor research, enhancing our understanding of tumor types [22]. However, previous bioinformatics analyses have seldom integrated these technologies, which could complement each other to yield more comprehensive insights. Single-cell sequencing enables high-throughput analysis of individual cells, uncovering the heterogeneity and subpopulation structures within tumors. In contrast, spatial transcriptomics reveals the spatial distribution patterns of cells within tissues by analyzing gene expression in tissue sections. This integrated approach will advance tumor research in a more detailed and profound direction, offering stronger support for future personalized therapies. In this study, we employed single-cell sequencing and spatial transcriptomics to characterize exosome-related genes in renal cell carcinoma, thereby gaining deeper insights into the potential role of MIOX in RCC development. Our findings indicate that, at the single-cell level, MIOX exhibits a significant heterogenous expression pattern in RCC, with spatial transcriptomic data revealing expression differences in specific cell subpopulations.

Our study has some limitations. First, we established and validated ferroptosis-related differentially expressed genes from the TCGA public database KIRC cohort, and further validation cohorts are needed to confirm the expression of these ferroptosis-related genes. Secondly, this study only included differentially expressed ferroptosis-related genes, while many important non-differentially expressed genes associated with ferroptosis and tumorigenesis were excluded. Finally, regarding whether ferroptosis can overcome immune therapy resistance in renal clear-cell carcinoma, there is a limited number of patients in the TCGA database with information on immune and targeted therapies and treatment outcomes; therefore, further exploratory cohorts with treatment information are needed.

Currently, there are a variety of bioreagents used in ferroptosis-related research. They can also be categorized by their modes of action.

As the small molecule inhibitors for reducing lipid peroxides, Ferrostatin-1 (Fer-1) was identified as a ferroptosis inhibitor by Dixon et al. in 2012, which was proved to inhibit RSL3 or erastin-induced ferroptosis. It has been shown that Fer-1 functions by inhibiting lipid peroxidation because of the primary aromatic amine [6]. It is known that β-Mercaptoethanol (β-ME) inhibits ferroptosis when System Xc− is blocked in the process of helping cells uptake cystine. In this process, β-ME reacts with cystine and forms a mixed disulfide. The mixed disulfide is transferred into cells via System L and produces cystine rapidly. Small molecular inhibitors that reduce iron levels, Deferoxamine (DFO) is another widely used iron chelator, which has been reportedly used to inhibit ferroptosis with a therapeutic effect on traumatic spinal cord injury [23].

Compared to other inhibitors, Ferrostatin-1 has demonstrated protective effects in animal models of organ damage, such as kidney and liver injuries, by preventing ferroptosis-induced tissue damage, thus highlighting its potential in therapeutic applications for organ protection. Ferrostatin-1 has been reported to have minimal toxicity in vivo, making it a safer alternative compared to other experimental inhibitors that might cause side effects.

In summary, proximal tubules are generally considered the origin of renal cancer, and the specifically expressed myo-inositol oxygenase (MIOX) gene may play a crucial role in the mechanisms underlying the occurrence and progression of RCC.

## Materials and methods

### Data collection

The mRNA data for 538 patients with clear-cell renal cell carcinoma, including detailed clinical and molecular information such as gender, age, grade, histological type, TNM categories, pathologic stages, neoadjuvant treatment history, and survival data, were obtained from the TCGA database (https://portal.gdc.cancer.gov) and used as the training cohort. Subsequently, batch correction was carried out using the SVA package. Ferroptosis-related genes were sourced from FerrDb (http://www.zhounan.org/ferrdb/index.html) and GeneCards (https://www.Genecards.org/). They were provided in Supplementary Table 1. Consequently, the 291 ferroptosis related genes were included in the analysis.

### Cell culture and reagents

The human RCC cell strains A498 and OSRC-2 were sourced from the American Type Culture Collection (Manassas, VA, USA) and propagated following suggested guidelines. All cells were confirmed to be free from mycoplasma contamination and were validated using short tandem repeat (STR) profiling before experimentation. The cells were maintained in MEM (Gibco) supplemented with 10% FBS (Gibco) and incubated at 37 °C in a 5% CO_2_ humidified environment.

### Identification of ferroptosis-related DEGs

To identify differentially expressed genes (DEGs) between tumor tissues and adjacent normal tissues, TCGA-KIRC data was analyzed using the R package “limma” within RStudio, applying a significance threshold of *p* < 0.05 and |log2FC| ≥ 1. The “ggrepel” package was then utilized to create a Volcano Plot for visualizing these DEGs. Ferroptosis-related DEGs were determined by intersecting the list of 291 ferroptosis-related genes with the 1290 DEGs identified.

### Functional analysis

The R package “pheatmap” was employed to illustrate the extent of variations within the datasets. For the functional annotation of 30 ferroptosis-related DEGs, gene ontology (GO) and Kyoto Encyclopedia of Genes and Genomes (KEGG) analyses were visualized using the “ggplot” R package.

### Protein-protein interaction (PPI) network construction

The construction of the protein-protein interaction (PPI) network was carried out using the STRING database (https://string-db.org/), which provides comprehensive information on protein interactions based on experimental evidence as well as predicted interactions. By incorporating the target genes into STRING, we were able to develop PPI networks utilizing this well-established online resource.

### Survival analysis

To assess the prognostic potential of the 30 genes, we conducted ROC curve analysis and survival analysis. Kaplan-Meier survival curves were generated to evaluate differences in overall survival (OS) between high-risk and low-risk groups, utilizing the “survival” and “survminer” R packages. Differences were quantified using the log-rank test, with a significance threshold set at *p* < 0.05. The univariate Cox regression analysis utilized normalized expression data for DE-FRGs along with overall survival (OS) time and OS status as input files. DE-FRGs with *p* values below 0.05 were selected as potential prognostic biomarkers.

### Western blot

RCC cells were lysed using RIPA buffer (Cell Signaling) supplemented with 1 mM PMSF (Beyotime) and a 1× protease/phosphatase inhibitor cocktail (Roche). Protein concentrations were measured with the bicinchoninic acid (BCA) assay kit (Beyotime). Each sample, containing 30 μg of protein, was then loaded onto a 4–20% SDS-PAGE gel.

For protein identification, gel-separated proteins were transferred to Immuno-blot polyvinylidene fluoride membranes (Roche, 0.22 μm pore size) using the Trans-Blot Turbo Transfer system (Bio-Rad). The membranes were blocked in TBS-T (tris-buffered saline with 0.1% Tween-20) containing 5% skimmed milk for 3 h at 37 °C. Following this, the membranes were incubated overnight at 4°C with primary antibodies diluted in primary antibody dilution buffer (Beyotime). After three washes with TBS-T, horseradish peroxidase-linked secondary antibodies (in TBS-T with 1% skimmed milk) were applied for 60 minutes at room temperature. Chemiluminescent signals were visualized using an enhanced chemiluminescence system (Millipore, Billerica, MA, USA).

### RNA isolation and qRT-PCR

RNA was isolated using the TRIzol agent (Takara Bro Inc, 9108) following the recommended guidelines from the producer. Using the Hiscript® III Reverse Transcriptase kit (Vazyme, R223-01), 1 μg of RNA specimens were transcribed in reverse to generate cDNA. qRT-PCR was conducted on the QuantStudio™ 3 Real-Time PCR Detection System with the aid of ChamQ Universal SYBR qPCR Master Mix (Vazyme, Q711-02). Using the 2−∆∆CT technique, the comparative expression of each gene was measured and standardized based on the internal reference, glyceraldehyde-3-phosphate dehydrogenase (GAPDH).

### Bioluminescence imaging

Mice were anesthetized with isoflurane before imaging commenced. Imaging began one minute after the injection of D-luciferin. Bioluminescence signals were measured using the region-of-interest feature in Living Image software (version 4.4).

### Orthotopic xenograft model

BALB/c nude mice were utilized to create an orthotopic xenograft model. Six-week-old mice were randomly divided into two groups (*n* = 6 per group). The mice were then anesthetized, and approximately 1 × 10^6^ OSRC cells mixed with 40% Matrigel (BD, San Jose, CA, USA) were injected into the left subrenal capsule. Tumor growth was monitored biweekly using the AniView100 in vivo imaging system (BLT, Guangzhou, China). After six weeks, the mice were euthanized, and tumor specimens were collected for further analysis.

### Immunohistochemistry

Paraffin-embedded sections from clinical specimens, murine tumor samples, and tumor tissue microarrays were first deparaffinized using xylene and then rehydrated through a series of graded ethanol solutions. Next, a 3% H_2_O_2_ solution was applied for 15 min to inhibit endogenous peroxidase activity. The samples were then treated with a boiling antigen retrieval buffer for 30 min. After this, they were incubated overnight at 4 °C with primary antibodies. Following the incubation, HRP-linked secondary antibodies were added, and detection was performed using DAB. Finally, hematoxylin was applied as a nuclear counterstain, and the sections underwent dehydration using graded alcohols and xylene. DAB positive score analysis was performed using ImageJ software with IHC Profiler. H-score was performed for a quantitative analysis of the immunohistochemistry results. The H-score is calculated by considering both the intensity of the staining and the percentage of positively stained cells in the tissue sample. The H-score typically ranges from 0 to 300, where a higher score indicates stronger and/or more widespread expression of the target protein.

### Processing of single-cell sequencing data

We analyzed single-cell RNA sequencing data utilizing the R packages ‘Seurat’ and ‘SingleR’ [24]. To maintain high-quality cellular data, we focused on genes that were expressed in at least three individual cells. Additionally, we excluded cells with gene counts below 200 or above 10,000, as well as those with fewer than 1000 counts and those where more than 20% of the genes were mitochondrial or ribosomal.

To correct for batch effects between cancer and adjacent normal samples, we used the ‘harmony’ R package [25]. To cluster the integrated data, we utilized the ‘FindNeighbors’ and ‘FindClusters’ functions, visualizing the resulting cell groups with UMAP or t-SNE techniques. To pinpoint genes uniquely expressed in each cluster, we performed Wilcoxon tests between pairs of clusters, leveraging the ‘FindAllMarkers’ and ‘FindMarkers’ functions from the ‘scran’ R package. The expression patterns of specific genes were illustrated using the ‘featureplot’ function [26]. Cell type annotations were derived from the original literature as well as data from the tumor single-cell transcriptome database TISCH (http://tisch.comp-genomics.org/). Quantitative comparisons between cell subgroups were based on the gene expression units (FPKM). The high-MIOX expression group and low-MIOX expression group were distinguished based on the median of the overall data (i.e., above and below the median).

### Processing of spatial transcriptome sequencing data

The data is analyzed in R using Seurat. UMI counts undergo normalization and scaling, with the most variable features identified through the “SCTransform” function. For unsupervised clustering analysis, dimensionality reduction is performed using “RunPCA.” The “FindNeighbors” and “FindClusters” functions are applied with default settings, focusing on the 30 most significant principal components. The ‘SpatialFeaturePlot” function is employed to visualize subgroups and gene expressions. The Institute of Molecular Biosciences at the University of Queensland has developed an integrated analysis tool called the stlearn package (https://github.com/BiomedicalMachineLearning/stLearn). This package utilizes gene expression data, tissue morphology, and spatial location information to initially classify cell types and then reconstruct the distribution of these cell types within tissues.

### Statistical analysis

All statistical evaluations were performed using R software (Version 3.6.2) or GraphPad Prism (Version 9.0.0). Unless stated otherwise, a two-sided p-value or an adjusted p-value below 0.05 was deemed statistically significant. Data sorting and analysis were carried out using R software. For continuous variables that met the criteria of normal distribution and equal variance, an independent sample t-test was employed; otherwise, the Wilcoxon rank-sum test was utilized. Data were gathered from at least three separate experiments and presented as mean ± standard deviation. For comparisons involving three or more groups, one-way analysis of variance (ANOVA) was utilized. To compare each group to a single control, Dunnett’s test was applied. For multiple paired comparisons with equal group sizes, either Tukey’s test or the least significant difference method was used.

### Study approval

Every procedure that involved the animals adhered rigorously to the directives established by the Institutional Ethics Committee of First Affiliated Hospital of Fujian Medical University. Samples of human RCC, encompassing tissue microarrays, were procured after receiving written consent from the involved individuals. The Ethics Committee of Hospital granted authorization for this research.

## Supplementary information

Figure. S1

Supplementary Table 1

uncropped blot

## Data Availability

All data are included in the manuscript. Raw data would be available upon request.
