# Supplementary material for: Integrated analysis of MIOX gene in prognosis of clear-cell renal cell carcinoma
Source: Cell Death Dis. 2025 May 8;16(1):368. doi: 10.1038/s41419-025-07698-7 (PMC12062366; doi:10.1038/s41419-025-07698-7)
Supplement: Supplementary file 1 — Figure. S1 [file 41419_2025_7698_MOESM1_ESM.docx]

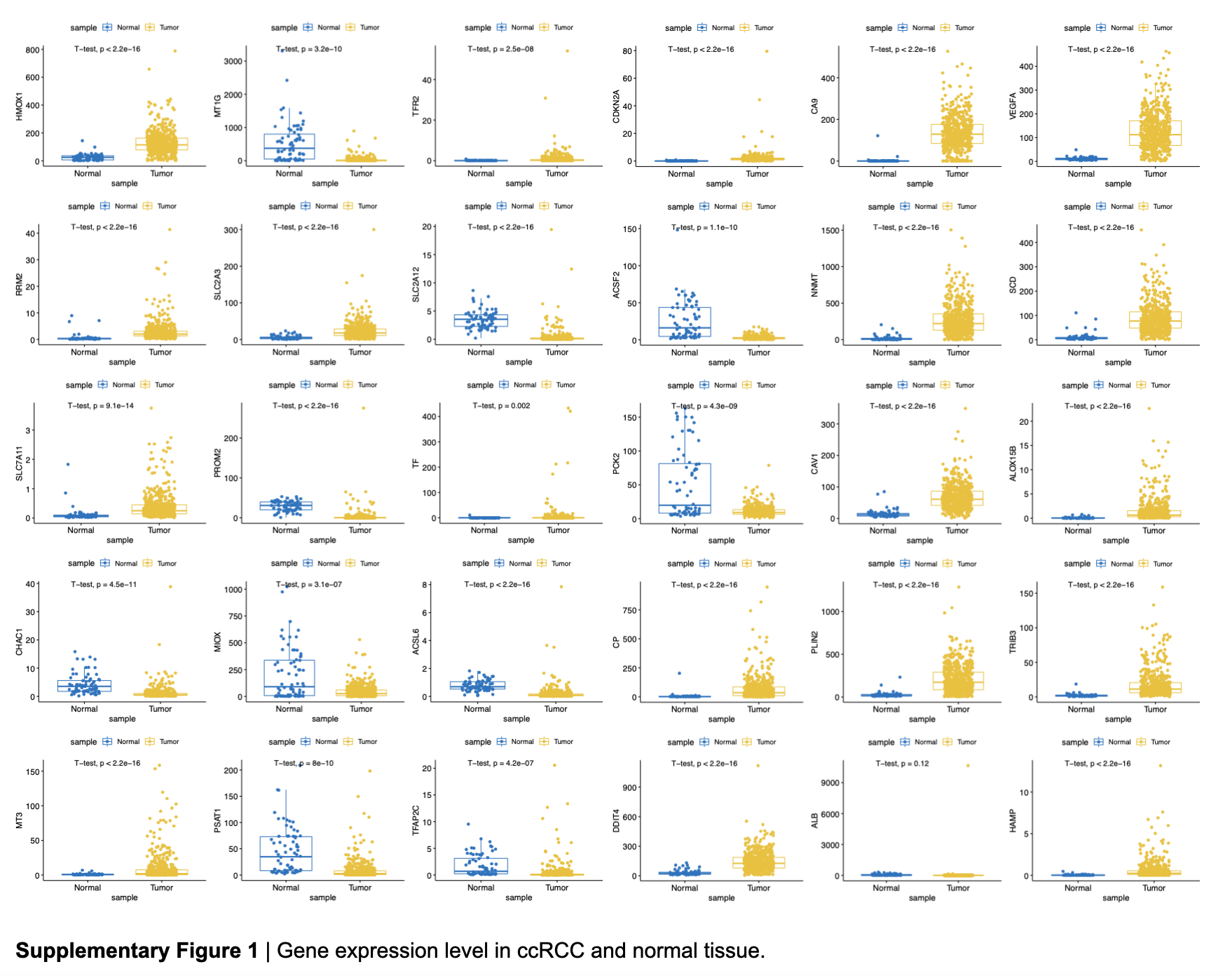


**Figure. S1 Expression of 30 ferroptosis-related DEGs in ccRCC and normal tissue**


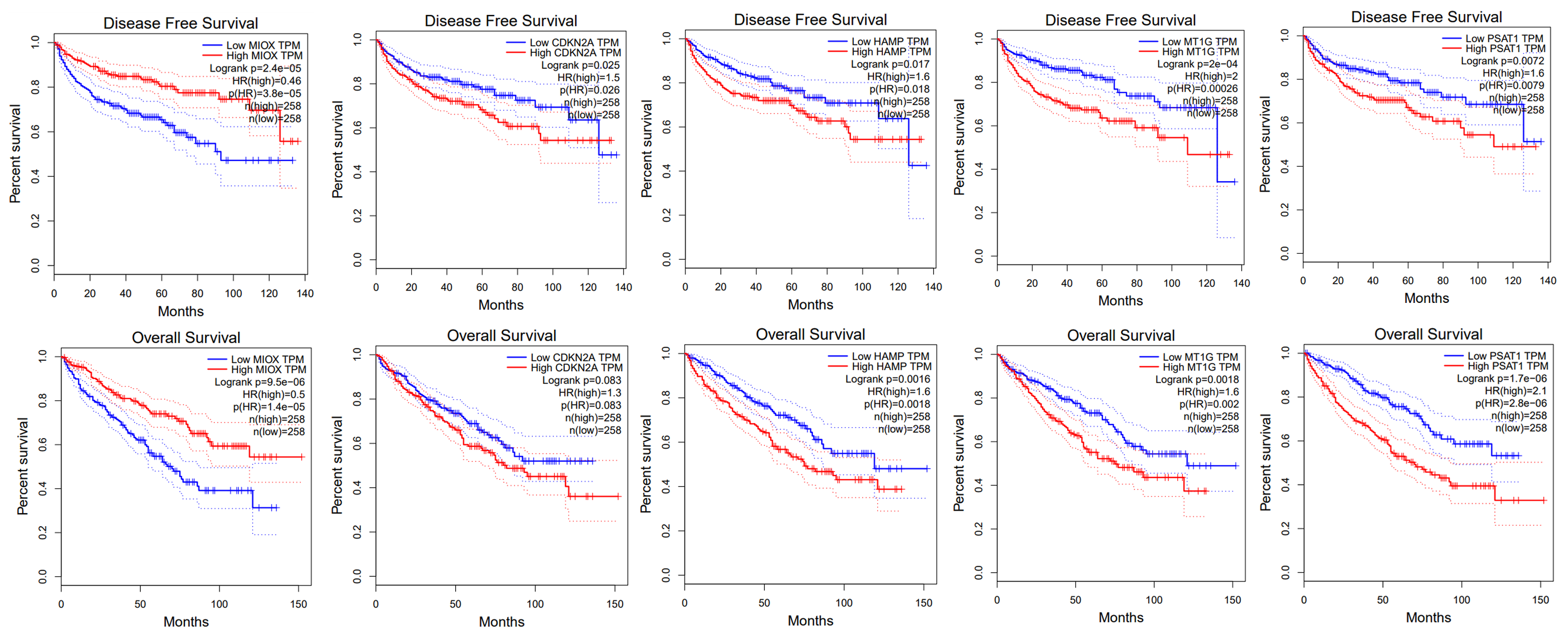


**Figure. S2 Kaplan-Meier curves for the DFS/OS of patients with high- and low-gene expression level in the TCGA cohort**


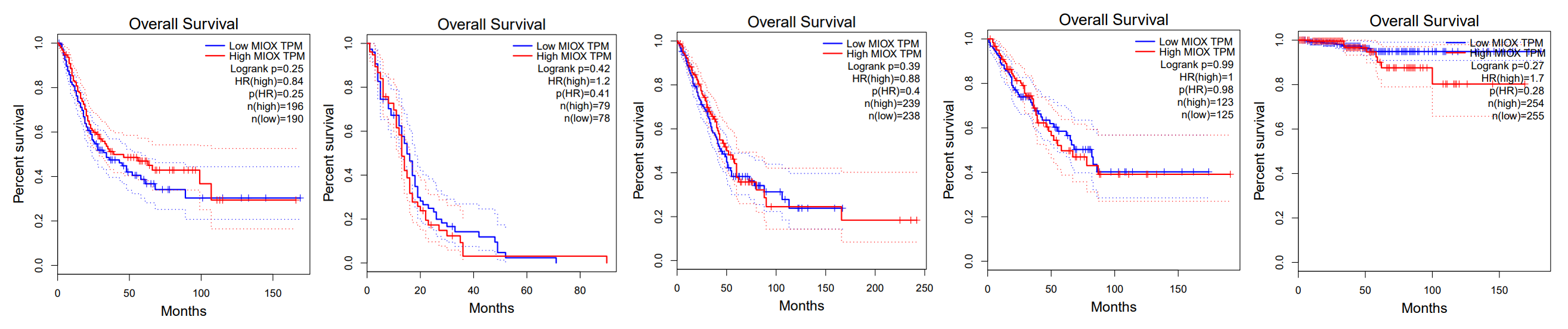


**Figure. S3 Kaplan-Meier curves for the OS of patients with high- and low-MIOX expression level in the TCGA -BLCA/GBM/LUAD/THCA cohort (left to right)**


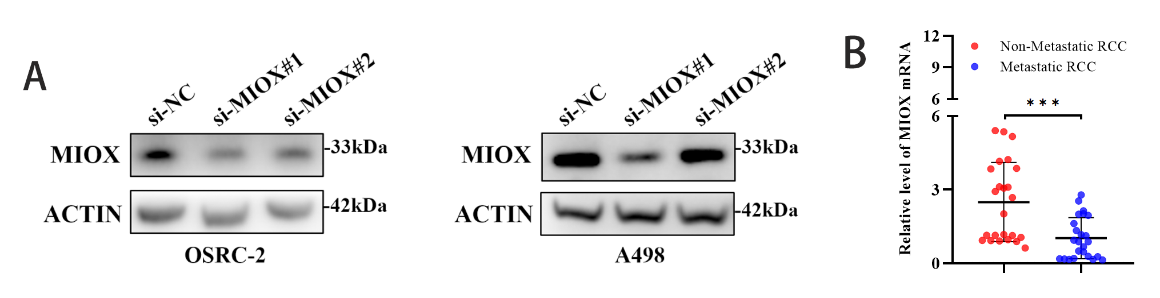


**Figure. S4 The expression of MIOX in cell lines and human ccRCC samples.**

A. Western blot was conducted to identify the efficiency of MIOX silencing

B. qRT-PCR was employed to detect the expression differences of MIOX in non-metastatic and metastatic ccRCC tissues.
