# Supplementary figures and images for: Integrated analysis of MIOX gene in prognosis of clear-cell renal cell carcinoma
Source: Cell Death Dis. 2025 May 8;16(1):368. doi: 10.1038/s41419-025-07698-7 (PMC12062366; doi:10.1038/s41419-025-07698-7)

## Slide 1
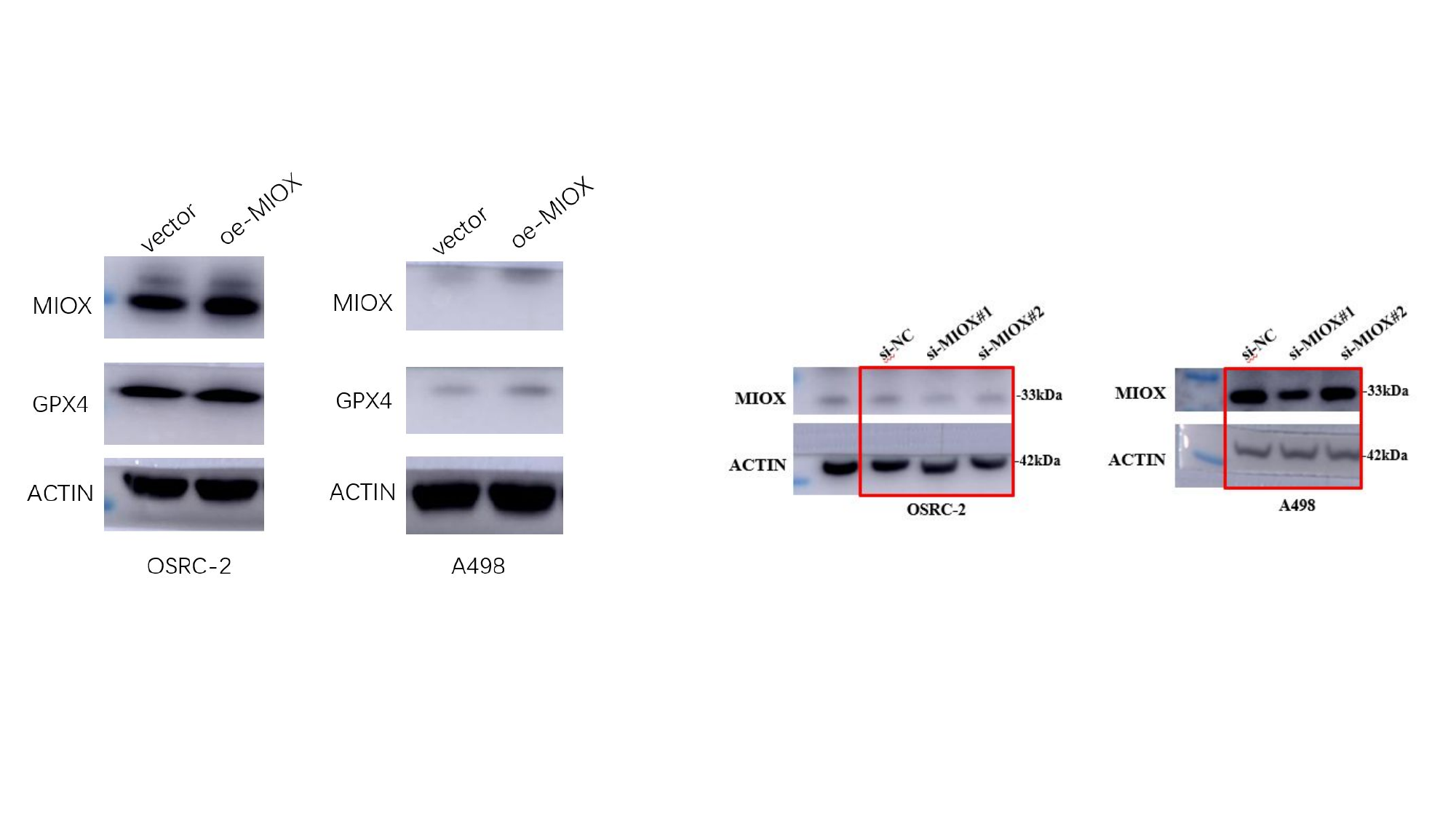

Supplement: Supplementary file 3 — uncropped blot [file 41419_2025_7698_MOESM3_ESM.pptx]
